# Qualia as query act, the phenomenology of predictive error coding

**DOI:** 10.3389/fpsyg.2025.1531269

**Published:** 2025-04-03

**Authors:** Herbert W. Harris

**Affiliations:** Independent Practitioner, Arlington, VA, United States

**Keywords:** predictive error, consciousness, phenomenology, active inference, qualia experience, intentionality, cognition

## Abstract

This paper explores the intersection of phenomenology and neuroscience to address foundational questions about consciousness, particularly the nature of qualia—subjective, ineffable contents of experience. Drawing on Thomas Nagel’s seminal inquiry into subjective experience and Husserlian phenomenology, we propose that phenomenological “What is it like?” questions can be integrated with neuroscientific models through predictive error coding (PEC). PEC reconceptualizes the brain as an active inference system, continuously generating and updating predictions about sensory inputs. We introduce the concept of “query acts” to describe the brain’s interrogative engagement with sensory information, linking intentionality in phenomenology with predictive mechanisms in neuroscience. By framing qualia as dynamic processes arising from query acts rather than static entities, we bridge the explanatory gap between subjective experience and objective inquiry. This interdisciplinary framework highlights structural parallels between noetic processes in phenomenology and PEC in neuroscience, providing a novel perspective on the emergence of conscious experience. Additionally, we explore the implications of query acts for clinical interventions in psychiatric disorders and the development of context-sensitive artificial intelligence systems. This synthesis fosters deeper integration of philosophical and scientific approaches to understanding the nature of consciousness.

## 1 Introduction

Thomas Nagel’s essay, “What is it Like to Be a Bat?” (Nagel, 1974), illustrates the challenges inherent in understanding consciousness from a first-person perspective. We can objectively describe the behavior of bats. We understand the neurophysiological mechanisms underlying their unique sensory processing. However, whether some aspect of the bat’s experience remains inaccessible to objective description remains. The answers we seek often point toward qualia—the subjective, ineffable contents of consciousness. When we inquire about what it is like to see a particular shade of red or to hear a specific musical note, we anticipate definitive, though private, responses. Qualia are considered the fundamental and irreducible facets of subjective experience. Yet, they gain significance only within the intentional context provided by the questions we pose. The “What is it like?” inquiries are central to defining and structuring our conscious reality.

Recent advancements in neuroscience have deepened our understanding of the mechanisms underlying conscious activity. However, this progress has unfolded independently of phenomenological methods emphasizing introspection and subjective experience. This divergence raises critical questions: How do concepts like qualia and intentionality integrate with neuroscientific models? Can phenomenology meaningfully inform neuroscience?

This paper proposes that phenomenological “What is it like?” questions can be conceptualized within the framework of predictive error coding (PEC). By examining the predictive processing of somatosensory and interoceptive signals, we will draw parallels to the noetic processes—the acts of consciousness—that constitute subjective experience. PEC systems do not merely transmit information; they actively query incoming data against predicted states, effectively asking, “Is this input like what we anticipated?” When predictions fail, error signals are generated, prompting top-down adjustments—a process reminiscent of Husserl’s concept of anticipatory apprehension in forming intentional objects (Husserl, 1982). In both phenomenological and neurobiological contexts, the mind adopts an active, interrogative stance toward information.

We introduce the term “query act” to encapsulate this process, emphasizing its biological basis and intentional character to provide a fruitful intersection between phenomenology and neuroscience. Ultimately, we suggest rethinking qualia in terms of query acts, offering a novel perspective that fosters interdisciplinary alignment.

## 2 Qualia

The concept of qualia is central to discussions in the philosophy of mind, cognitive science, and neuroscience, serving as a focal point for debates about the nature of consciousness and subjective experience. Qualia refer to the intrinsic, subjective qualities of conscious experience—the “raw feels” or the phenomenal aspects of the mind (Lewis, 1929). Examples include the redness one experiences when seeing a sunset, the bitterness tasted in dark chocolate, or the sharp pain felt when touching a hot surface.

Thomas Nagel’s influential essay “What Is It Like to Be a Bat?” (1974) revitalized interest in the subjective aspect of consciousness. Nagel argues that an organism has conscious experience if and only if there is something it is like to be that organism. He emphasizes that subjective experience is essentially connected to a specific point of view, which cannot be fully apprehended through objective, third-person descriptions. Using bats—creatures with a sensory apparatus vastly different from humans—as an example, Nagel illustrates how we can study the neurophysiology of bats and understand echolocation scientifically. Yet, we cannot know what it is like for the bat to experience the world this way. This illustrates the explanatory gap between objective accounts of brain processes and the subjective qualities of experience—qualia.

The relationship between qualia and “What is it like?” questions reveals the deep connection between subjective experience and intentionality. In phenomenology, intentionality refers to the directedness of consciousness toward objects or states of affairs (Husserl, 1982). When we ask, “What is it like to see red?” we are directing our consciousness toward the qualitative experience associated with perceiving red—a quale. Qualia are deeply subjective and resist complete articulation. Their ineffable nature means they cannot be fully conveyed through language or objective descriptions. This is why simply providing answers to “What is it like?” questions does not capture the essence of qualia.

We will argue that understanding qualia in terms of “What is it like?” questions may bridge the gap between subjective experience and objective inquiry. This view emphasizes the importance of first-person perspectives while recognizing that questions of this form have concrete embodiments in the neural circuitry of PEC.

## 3 Phenomenological accounts of perception

Phenomenology, founded by Edmund Husserl in the early 20th century, represents a philosophical movement dedicated to describing the structures of experience as they present themselves to consciousness. This approach eschews theory, deduction, or assumptions from other disciplines to study phenomena—the appearances of things—as they are experienced from the first-person point of view (Husserl, 1982).

At the heart of Husserl’s phenomenology lies the concept of intentionality, originally developed by Brentano (1995) and subsequently adopted by Husserl. Intentionality refers to the mind’s capacity to be directed toward or about something—every mental act is an act of consciousness of an object. This “object” need not be a physical thing; it can be an idea, a feeling, or any content toward which consciousness is directed.

Husserl dissected the structure of intentional acts into two correlated components: noesis and noema. The noetic component represents the subjective aspect of the intentional act—the mental activity or process by which the object is apprehended. It encompasses various modes of consciousness, such as questioning, judging, imagining, and remembering. The noematic component is the intentional content or the object as it is meant in the experience. The noema includes the object’s perceived properties and how it is presented to consciousness (Husserl, 1982).

The term hyletic refers to the raw, sensory data of experience—the sensations, feelings, and sensory impressions that serve as the substrate for intentional acts (Husserl, 1997). These data are passively received and provide the content structured by the mind in the process of perception. This structuring process involves anticipatory apprehension (Vorgriff), the notion that perception involves an active, forward-looking component. Consciousness does not merely register sensory inputs but anticipates what will come based on prior experience and context (Husserl, 1991). This anticipatory aspect allows for the seamless flow of perception, filling in gaps and making sense of incomplete information. Husserl introduced the concept of horizon to explain how we experience objects and events within a broader context. The horizon is a dynamic context that shapes all experiences, providing the backdrop against which objects and events are meaningfully constituted in consciousness.

These phenomenological insights provide a valuable framework for understanding how intentionality might be embodied in neurobiological systems. The parallels between phenomenological accounts and predictive coding models are striking. Just as Husserl’s consciousness anticipates and interprets sensory data, predictive coding models propose that the brain generates predictions about incoming stimuli and updates these predictions based on prediction errors. The noetic processes can be likened to computational mechanisms that construct the intentional content (noema) from sensory inputs. At the same time, the concept of horizon aligns with the brain’s use of contextual information and prior knowledge to interpret sensory inputs.

**Figure 1:**
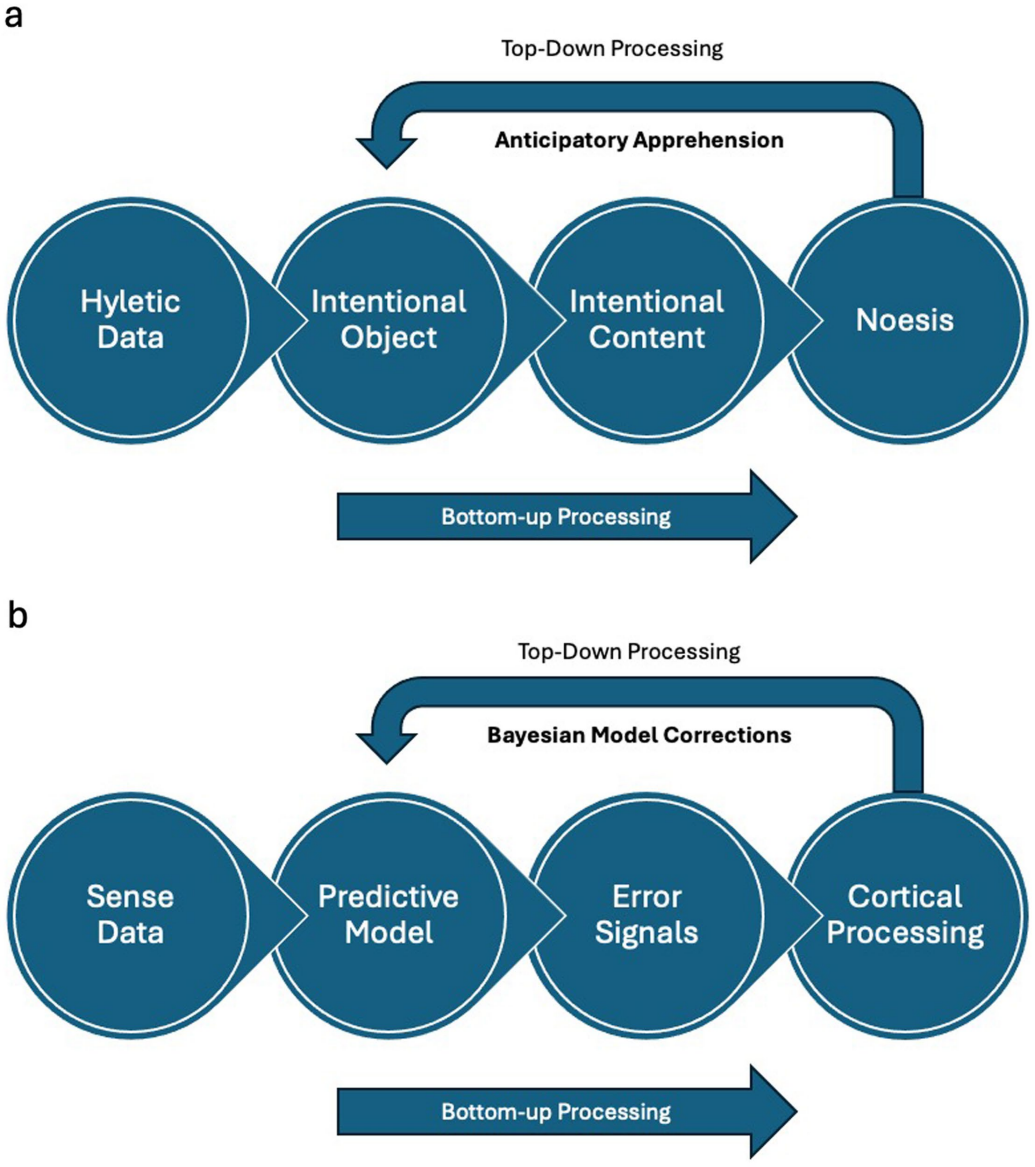
Parallel architectures in phenomenological experience and predictive processing. **(a)** Phenomenological framework of conscious experience. The diagram illustrates the hierarchical structure of conscious experience as conceptualized in Husserlian phenomenology. At its foundation lies hyletic data—the raw sensory impressions that enter consciousness, such as patterns of light or sound waves, forming the basic building blocks of experience. These uninterpreted sensory materials flow upward through successive levels of processing and interpretation. From this foundation, the system constructs intentional objects, representing the first level of organized perception. Here, the mind begins to recognize distinct entities and forms, integrating the raw sensory elements into coherent objects. These objects then develop into intentional content, where perception gains meaning through contextualization and association with memories and learned patterns. At the highest level, noetic acts represent sophisticated conscious processes that actively interpret and create meaning from the lower-level information. This hierarchical arrangement is animated by two essential flows: a bottom-up process (indicated by blue arrows) that carries sensory information upward through successive levels of interpretation, transforming raw sensation into meaningful experience, and a top-down process (shown by red arrows) that embodies anticipatory apprehension (Vorgriff), demonstrating how higher-level understanding actively shapes lower-level perception. **(b)** Neural architecture of predictive error coding. This diagram reveals how the brain implements predictive error coding through multiple levels of neural processing. The journey begins with sensory input—raw neural signals from sensory organs that provide the initial data for processing. As this information ascends through the neural hierarchy, it encounters successive levels of increasingly sophisticated processing. The second level generates error signals by comparing incoming sensory data against predictions, creating a crucial feedback mechanism for updating the brain’s internal models. These models, represented at the third level, maintain dynamic predictions about expected sensory input, continuously refined by incoming error signals. At the apex, higher cortical processing integrates this information into abstract representations and coordinates predictions across the entire system. The diagram shows two critical information flows: bottom-up processing (marked by purple arrows) carries prediction error signals upward, indicating how sensory mismatches drive model updates, while top-down processing (shown in green arrows) represents how predictive model updates cascade downward to shape lower-level processing. This bidirectional flow creates a continuous dialogue between sensory evidence and predictive models, embodying the brain’s active inference framework. The parallel organization of these two frameworks—phenomenological and neural—reveals their fundamental structural similarities, supporting the paper’s central argument that conscious experience and neural prediction share core organizational principles. Both systems demonstrate hierarchical processing, bidirectional information flow, and active interpretation of sensory data, suggesting that query acts may indeed represent a common mechanism underlying both conscious experience and neural processing.

By drawing on these phenomenological concepts, we can explore how predictive error coding systems may embody a form of intentionality. The brain’s predictive mechanisms could embody intentional acts that construct and update models of the world, much like Husserl’s consciousness constitutes objects through intentionality. Parallels between the noetic process and PEC are illustrated in Figure 1. While PEC may not underlie all conscious perception, it offers promise as a bridge between phenomenology and neuroscience. It suggests that the structures of conscious experience have neurobiological correlates in the brain’s predictive architecture, opening avenues for interdisciplinary research that enriches our understanding of perception, cognition, and the nature of consciousness.

## 4 Predictive coding

Predictive coding is a theoretical framework that conceptualizes the brain as a predictive machine. It continuously generates and updates models of the environment to minimize the discrepancy between expected and actual sensory inputs, which is known as prediction errors. This approach has gained prominence in neuroscience for its ability to unify diverse cognitive and perceptual phenomena under a common computational principle.

The roots of predictive coding trace back to the 19th century with Hermann von Helmholtz’s notion of unconscious inference in perception (Helmholtz, 1867). Helmholtz proposed that the brain interprets sensory data by inferring the most probable causes of stimuli based on prior experiences. In the mid-20th century, Claude Shannon’s information theory (Shannon, 1948) laid the groundwork for understanding efficient coding and data compression. Building on this, Elias (1955) introduced predictive coding in signal processing, demonstrating how future inputs could be predicted from past data to reduce redundancy in communication systems. The application of predictive coding to neuroscience emerged prominently with the work of Rao and Ballard (1999), who developed a hierarchical model of the visual cortex. Friston further expanded the framework, integrating it with the free energy principle to explain a wide array of neural and cognitive processes (Friston, 2005; Friston, 2010).

The framework has demonstrated remarkable explanatory power across various domains of brain function. In sensory processing, predictive coding accounts for extra-classical receptive field effects in vision, such as contextual modulation and perceptual illusions, by explaining how the brain integrates contextual information to predict sensory inputs (Alink et al., 2010). It elucidates phenomena like repetition suppression and mismatch negativity through adaptive updating of predictions (Summerfield et al., 2008; Stefanics et al., 2014). In addition, the framework explains auditory scene analysis, including the cocktail party effect and speech perception in noisy environments, as demonstrated by Arnal and Giraud (2012) and Heilbron and Chait (2018). For somatosensation, predictive coding accounts for sensory attenuation during self-generated movements, proposing that expected sensory consequences of actions are discounted, which helps differentiate between self and external stimuli (Brown et al., 2013; Palmer et al., 2016).

In higher cognitive processing, predictive coding models suggest that attention enhances the precision of prediction errors for relevant stimuli, effectively allocating computational resources to important information (Feldman and Friston, 2010; Aitchison and Lengyel, 2017). The framework extends to interoception and emotion, explaining how the brain predicts physiological states and how discrepancies contribute to emotional experiences and affective disorders (Barrett and Simmons, 2015; Seth and Friston, 2016). In consciousness studies, predictive coding suggests that conscious experience and the sense of self emerge from the brain’s predictive models of its own states and interactions with the environment (Hohwy, 2013; Limanowski and Friston, 2020).

Dysfunctions in predictive processes have been implicated in various psychiatric conditions. Altered precision weighting of prediction errors may underlie hallucinations in schizophrenia, sensory sensitivities in autism, and various symptoms in depression (Corlett et al., 2019; Lawson et al., 2017; Smith et al., 2021). This clinical relevance highlights the framework’s potential therapeutic applications.

## 5 Bridging qualia and predictive error coding: the query act

Traditional views of sensory processing often depict the brain as a passive recipient of external stimuli, constructing perceptions based solely on incoming sensory data. However, the predictive coding framework reconceptualizes the brain as an active inference machine (Friston, 2010). In this model, the brain continuously generates predictions about sensory inputs based on internal models of the world and compares these predictions to actual sensory inputs, effectively engaging in an ongoing process of hypothesis testing (Clark, 2013). When sensory inputs match predictions, the brain’s internal models are reinforced. Conversely, when there is a mismatch—known as a prediction error—the brain updates its models to better account for new information. This iterative process reflects an active, interrogative stance toward sensory information, where the brain is not merely processing inputs but actively questioning and interpreting them (Seth, 2015).

In phenomenology, particularly in Husserl’s framework, perception is not a passive reception but an active constitution of experience through noetic processes (Husserl, 1982). Consciousness engages in intentional acts that interpret and give meaning to sensory data, forming coherent intentional objects within a contextual horizon. The parallels between PEC and noetic processes are striking. Both systems involve anticipatory mechanisms. In PEC, the brain predicts sensory inputs, and in phenomenology, consciousness engages in anticipatory apprehension (Vorgriff), which is expecting certain experiences based on past interactions. Both frameworks emphasize error detection and adjustment, where prediction errors in PEC prompt model adjustments (Friston et al., 2011), while in phenomenology, consciousness revises its interpretations to reconcile discrepancies.

To encapsulate the active, interrogative nature of both PEC systems and phenomenological consciousness, we introduce the term “query act.” A query act represents the process by which the brain or consciousness actively interrogates sensory inputs against predictions or expectations, embodying intentionality in both biological and phenomenological contexts. In neuroscience, a query act reflects the brain’s predictive mechanisms that generate hypotheses about sensory inputs and test them against actual data, resulting in prediction errors and model updates (Seth and Friston, 2016). From a phenomenological perspective, a query act embodies intentionality—the directedness of consciousness toward an object or content (Husserl, 1982). Central to the query act is the notion of questioning or interrogating inputs, where both the brain and consciousness are seen as asking, “Is this input like what we anticipated?”

To appreciate the significance of query acts as embodiments of intentionality, consider the role of interrogative statements in creating context and meaning in language. A traditional view in analytic philosophy holds that the meaning of a proposition, such as “The cat is on the mat,” hinges on its successful reference to entities and states in the world. However, in ordinary language, much of the proposition’s meaning in ordinary language depends on context. The proposition may be a response to the question, “Where is the cat?” or “What is that on the mat?” creating different contexts. We may think of query acts as prelinguistic questions embodied by neuronal processes. In language, the question’s nature shapes the proposition’s meaning. Similarly, when PEC circuits embody query acts, they shape the nature of conscious experience.

By reconceptualizing qualia in terms of query acts, we shift from seeing qualia as static, atomistic entities to a view of qualia as dynamic processes that result from active interrogation of sensory inputs. Qualia emerge from the brain’s continuous querying and updating of sensory predictions not as fixed entities but as active processes emerging from ongoing interactions between predictions and inputs (Clark, 2013; Seth, 2015). Furthermore, qualia are shaped by the intentional content of consciousness, influenced by context, expectations, and prior experiences. Seeing the color red happens when the brain, presented with a certain visual stimulus, engages in an active query that asks, “What is it like to see red?”

This reconceptualization offers several advantages. It provides interdisciplinary alignment by framing qualia as products of query acts, bringing into line phenomenological insights and neuroscientific models. On this interpretation, qualia retain their private nature insofar as individual differences in experience, predictive models, and query acts constitute the subjectivity of qualia. Therefore, qualia have a subjectivity that resists eliminative accounts like Dennett (1988, 1991), but they become more accessible to neuroscientific understanding. This approach identifies a crucial contact point between neuroscience and phenomenology, enabling both disciplines to progress and inform each other.

The query act framework opens promising avenues for both clinical applications and artificial intelligence development. In clinical settings, hallucinations and delusions may arise from qualitatively mismatched queries—the brain asking inappropriate questions of sensory data. For example, synesthetic experiences where individuals perceive colors in response to sounds could result from the brain erroneously querying, “What color is this sound?” This suggests that therapeutic interventions might focus on restructuring these fundamental query acts rather than just addressing their symptomatic manifestations. Similarly, certain forms of psychosis might reflect disrupted predictive processes where the brain poses queries that generate systematic misinterpretations of social and sensory information. For artificial intelligence development, the query act model suggests new architectures for neural networks that explicitly incorporate question-asking mechanisms. Rather than simply processing input data through fixed pathways, AI systems could be designed to actively query their inputs, leading to more flexible and context-aware AI systems that better mirror human cognitive processes.

## Data Availability

The original contributions presented in the study are included in the article/supplementary material, further inquiries can be directed to the corresponding author.
